# On the Use of Veratrum Viride in Pneumonia and Certain Forms of Fever

**Published:** 1874-01

**Authors:** Wm. L. M. Harris


### CORRESPONDENCE.

GREENESBORO, GA., January 8, 1874.

*Editors Southern Medical Record:*

The *"red cross"* I do not like to see on a journal of as much real value as is the RECORD. Enclosed please find five dollars, which you will place to my credit. You are publishing the cheapest journal in the country, and one among *the very best.* The arrangement, as well as the management, is admirable, and I know of no medical journal so completely adapted to the wants of the active practitioner.

Let every doctor who is a subscriber make it his business, and feel it to be his duty, never to meet another doctor without inquiring of him whether he takes *our* journal, and if he does not, secure his name and *money* at once, before letting him go. This shall be my rule.

I have long intended to send you some short "communications." I place at your disposal the following:

### *ON THE USE OF VERATRUM VIRIDE IN PNEUMONIA AND CERTAIN FORMS OF FEVER.*

When veratrum viride was first introduced to the profession, it was hailed as a substitute for the lancet, a thing long desired. It has not proven to be a complete substitute, but one thing is certain: the lancet is very much less used now than before that time, especially in the formidable and ever-to-be-dreaded disease *pneumonia,* for which I regard veratrum almost, if not quite, a specific. Let no reader at this point hold up his hands in holy horror, but read on. I do not belong to that class of physicians who believe that pneumonia is a disease not amenable to treatment—that it is self-limited, or runs a definite course, and terminates well or ill by *chance.* Who, that is well informed, doubts that many cases of true pneumonia have been "cut short," cured, by a *free and full blood-letting in the first stage,* or by the BOLD use of tartar emetic? What was, or is, accomplished by the use of the lancet or the use of tartar emetic in pneumonia? Why did, or do, we abstract blood? Was it not to "control the circulation"?—to "control the pulse or the heart's action"? This was, and is, the avowed object of the treatment; and to make the bleeding effectual, it must be carried to the point of *full* or *approaching* syncope. Mark this. And why? Not, certainly, to take from the body the cause, the agent, or subtle poison, whatever it might be, producing the disease. But to unload and relieve the congested and oppressed capillaries of the inflamed part or tissue—*first*, by reducing the amount of the circulating fluid; and *secondly*, by *stopping* for a time, and nearly stopping for a considerable time, the contractions or pulsations of the heart—thereby preventing an aggravated pumping or forcing of blood there through obstructed vessels. Thus might these capillaries, *by rest*, through their own well-established elasticity and contractibility, and the gentle pressure of slow and feeble pulsations from the heart, be relieved, and the trouble overcome. This I consider to be the philosophy of blood-letting in pneumonia, and *true* philosophy. I make no war upon the lancet, but do hold that *veratrum* is THE remedy in pneumonia, and propose to prove it.

The average number of beats per minute of the heart in a healthy adult is reckoned at seventy-five. Each one of these beats or pulsations sends a quantity of blood to and from the lungs. Without stopping to consider that very wonderful net-work of vessels and tissues which constitute the lung substance, it may be sufficient to say, that it is extensive and delicate, and that every drop of blood in the body *must pass* through it in "one minute of time," in order that it may be aerated, oxygenated or purified, and fitted for the sustenance of life. This is not true of any other organs or parts of the body. Every other organ and part is supplied by its own special vessel or vessels, conveying and giving whatever of the general circulating mass—the pabulum vitæ—that may be necessary for nutrition and secretion and no more, but the *whole* must pass through the lungs. Now when inflammation takes place here, and the pulse runs up to one hundred and forty or one hundred and fifty per minute, how very great must be the tax, the tension, the *strain* upon these vessels!

By simple mechanics, it would appear that any means by which this strain could be diminished or removed, would be the remedy to be used. As already stated, the lancet was used for this purpose, and upon correct reasoning; but its effects were of short duration, and soon the blood-letting had to be repeated and again repeated, and could the bleeding have been continued sufficiently long, the difficulty would have been overcome. Stay the heart's action; make it beat forty or fifty times to the minute, instead of one hundred and forty or one hundred and fifty times, and how great must be the relief given to the inflamed part! Now, continue this for "hours and hours" together, and it seems plain to me that you have done a great deal in aiding nature to overcome the disease. This, I know, is old doctrine, but in my humble judgment it is good doctrine. Now, I hold that by the use of veratrum, this object can be *certainly* and *safely* accomplished in every case. It *never fails* to reduce the heart's action. By its use you may lower the pulse to any number of beats you may choose, and keep it there as long as you may desire.

Now a word as to its *administration.* Physicians fail to realize the good effects of this most valuable medicine, because *they do not give enough of it.* When called to a case of pneumonia, I begin with from seven to ten drops, according to the character of the fever and the pulse, of Norwood's tincture every three or four hours, increasing the dose one or two drops each time *until the pulse is reduced to such degree as to cause sickness of the stomaeh or vomiting,* which never occurs till the pulse is reduced considerably below the natural standard. Whenever this condition of things is brought about, the pain, the distress of breathing, the cough, every difficulty is relieved, and by keeping up the action of the medicine judiciously for a sufficient length of time, we positively choke the inflammation out. Sometimes this sickness of the stomach becomes *alarming* to those who know nothing of its nature. The patient is pale and shrunken, and bathed in a frightful perspiration, and seems to be sinking—a state of things very much like what the *Thomsonians* call "the alarms," produced by the too free use of lobelia. This state is a little too much of a good thing, but for it we have an infallible remedy, and *the patient never fails to get well.* At least, such is my experience. The antidote is morphine or laudanum in proper doses, given in brandy or whisky, and repeated, if necessary. I would like here to give a few cases illustrative of what I have written, but find that my communication is already too long, and must conclude with "*more anon.*" WM. L. M. HARRIS.

